# Panmixia in a Fragmented and Unstable Environment: The Hydrothermal Shrimp *Rimicaris exoculata* Disperses Extensively along the Mid-Atlantic Ridge

**DOI:** 10.1371/journal.pone.0038521

**Published:** 2012-06-05

**Authors:** Sara Teixeira, Ester A. Serrão, Sophie Arnaud-Haond

**Affiliations:** 1 Ifremer – Centre de Brest, Departement “Etude des Ecosystèmes Profonds” - DEEP, Brest, France; 2 Centre of Marine Sciences, CIMAR, University of Algarve, Campus of Gambelas, Faro, Portugal; Université Paris Sud, France

## Abstract

Dispersal plays a fundamental role in the evolution and persistence of species, and especially for species inhabiting extreme, ephemeral and highly fragmented habitats as hydrothermal vents. The Mid-Atlantic Ridge endemic shrimp species *Rimicaris exoculata* was studied using microsatellite markers to infer connectivity along the 7100-Km range encompassing the sampled sites. Astonishingly, no genetic differentiation was found between individuals from the different geographic origins, supporting a scenario of widespread large-scale dispersal despite the habitat distance and fragmentation. We hypothesize that delayed metamorphosis associated to temperature differences or even active directed migration dependent on physical and/or chemical stimuli could explain these results and warrant further studies on adaptation and dispersal mechanisms.

## Introduction

Hydrothermal vents are extreme habitats that are distributed worldwide in association with volcanic and tectonic events. The fluids emitted at vent sites are geo-thermally heated up to several hundred degrees Celsius and enriched with minerals, metals and reduced chemicals, yet, they support highly populated communities of organisms that are specifically associated to these ecosystems. Most of the vent-restricted species have developed specific adaptations to extreme conditions and close relationships with chemoautotrophic microorganisms that are the food web’s primary producers in the absence of photosynthesis.

**Table 1:** Details of vent sites analysed.

Population	GPS coordinates	Depth	Cruise
Rainbow	36°08′44′′N 34°00′02′′W	2200 m	MoMAR; ROV Victor6000; RV Pourquoi pas?
TAG	26°02′34′′N 44°54′00′′W	3650 m	EXOMAR; ROV Victor6000; RV L’Atalante
Logatchev (Irina2)	14°44′62′′N 46°35′59′′W	2860 m	SERPENTINE; ROV Victor6000; RV Pourquoi pas?
Ashadzé (SE1)	12°50′71′′N 44°54′47′′W	3200 m	SERPENTINE; ROV Victor6000; RV Pourquoi pas?
SouthMAR	4°47′S 12°22′W	3048 m	M64/1; ROV Meteor/Quest

Detailed information of geographic coordinates, cruise and depth of the hydrothermal vents sampled along the Mid-Atlantic Ridge.

Hydrothermal vent species fully represent the paradigm of larval dispersal. The high level of endemism observed among [1], and within [2] hydrothermal vent biogeographic regions, supports a dominant strategy of scarce large-scale dispersal and high self-recruitment in these highly fragmented ecosystems. However, the frequent volcanic and tectonic events that often lead to the destruction and/or birth of these habitats [3], [4], suggests the capacity of hydrothermal species to maintain large-scale dispersal ability, allowing them to persist in such ephemeral habitats. A surprisingly rapid re-colonization was indeed suggested after an event of extinction – reactivation of a Pacific vent [4].

The remoteness of hydrothermal vents renders the assessment of migration difficult, and calls for indirect population genetic methods to infer rates and patterns of connectivity in the depth of the ocean. However, vent habitats scattered along a ridge, due to their instability and high extinction rates, might be considered as metapopulation(s), where the migration-drift equilibrium might never be reached [5].

Phylogeographic studies to date suggest the existence of barriers to dispersal along oceanic ridges, the degree of realized dispersal and the strength of the barrier vary to a certain extent according to life history traits, such as egg and larvae characteristics [6], [7]. Along the East Pacific Rise (EPR), three main dispersal barriers have been described, near the equatorial region, the Galapagos triple junction and the Easter microplate (see review [8]). Most genetic studies on vent organisms to date were conducted on the EPR. The Mid-Atlantic Ridge (MAR), although much less studied, seems to also have some dispersal filters, for *Bathymodiolus* mussels [9], [10], for which a semi-permeable barrier was found around the Broken Spur vent field, where these mussels are hypothesised to have hybridized for a long time [11], and between 14 and 23°N for their commensal polychaete [12]. These studies reveal a general capacity for dispersal of vent organisms, but also the presence of potential dispersal barriers.

In the Mid-Atlantic Ridge the most ubiquitous species is the Bresiliid shrimp *Rimicaris exoculata*, which is often present in high-density swarms (>1500 individuals/m^2^) around high-temperature sulphide chimneys at deeper hydrothermal vent sites [13], [14] and is considered endemic to those ecosystems. Genetic studies have determined two morphotypes in *R. exoculata*, corresponding to the adult and juvenile stages [15], [16]. These differ on the shape of the dorsal organ (oval on juveniles), behaviour (juveniles aggregate in cooler vent effluents) and colour, which is associated to their lipid composition; orange juveniles exhibit photosynthetically derived lipids whereas grey adults have lipids from bacterial origin [17], [18], [19]. The nutrition of adult *R. exoculata* is thought to originate mainly from specialized chemosynthetic epibionts present in their gill chamber and gut ([20], [21] and references therein).

**Figure 1:**
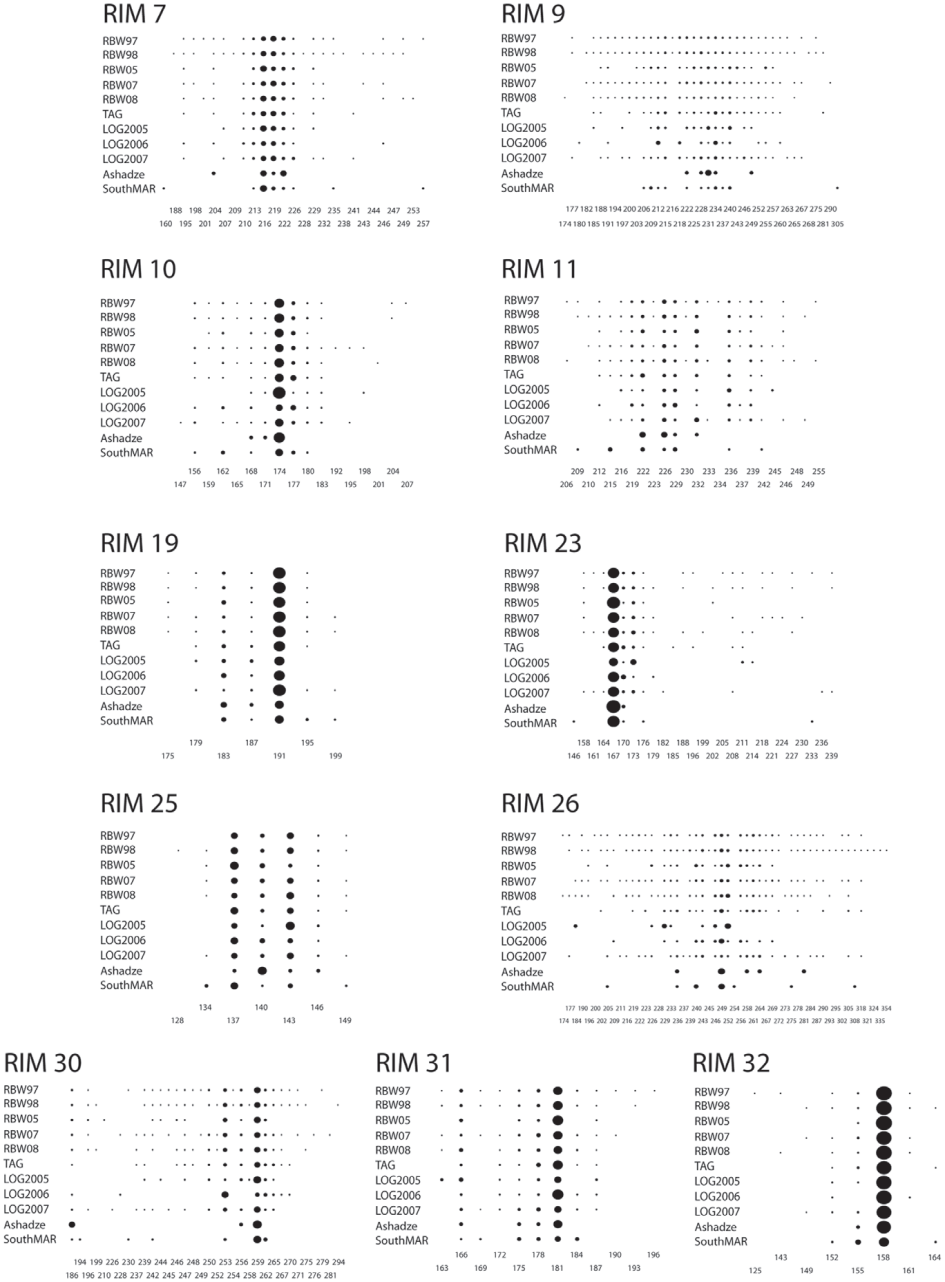
Allele distributions. Distribution of the alleles found for each locus at the different hydrothermal vent sites sampled along the Mid-Atlantic Ridge and for all sampling dates. For locus Rim 9 and Rim 26 not all alleles are represented numerically underneath the respective graph, however, the allelic distribution is represented in the graphs for all alleles found. Population codes: RBW- Rainbow; LOG- Logatchev.

Previous genetic studies of *Rimicaris exoculata* have used allozymes [15], [16] and mitochondrial DNA markers [22]. The allozyme markers revealed low genetic diversity and no genetic differentiation between the two studied populations, with an estimated migration of over 100 migrants per generation [15]. High levels of genetic diversity, low genetic differentiation and signatures of recent population expansion on 5 hydrothermal vent sites were discovered using COI mitochondrial DNA [22]. Recent and current patterns of connectivity between vent sites at large scales are still largely unknown, requiring highly polymorphic markers and widespread biogeographical sampling. The most used genetic markers, mitochondrial DNA (mtDNA) sequences, mainly provide information about historical processes for matrilineal relationships [23]. The mitochondrial genome is more prone to genetic drift than nuclear markers [24] but may not be sufficiently variable to resolve contemporary gene flow and is potentially affected by selective processes [25]. Microsatellites, in contrast, are capable of providing estimates of contemporaneous gene flow because of their high levels of polymorphism, biparental inheritance and frequently selective neutrality [26]. Polymorphic microsatellites are therefore expected to provide strong discriminatory power for resolving spatial and temporal population structure.

**Table 2:** Descriptive statistics of all sampled locations.

Population	n	*A*	*A_rich_*	*H*_E_	*H*_O_	*F*_IS_	*F*_IS_ using 7 loci
Rainbow 1997	149	16.73	4.75	0.68	0.62	0.096***	0.059***
Rainbow 1998	166	17.91	4.75	0.68	0.62	0.095***	0.071***
Rainbow 2005	28	8.73	4.52	0.66	0.58	0.118***	0.069*
Rainbow 2007	108	15.09	4.81	0.69	0.66	0.055***	0.018
Rainbow 2008	102	14.36	4.73	0.69	0.62	0.094***	0.027
TAG	39	10.82	4.61	0.69	0.67	0.039	0.022
Logatchev 2005	13	7.09	4.72	0.70	0.61	0.131**	−0.026
Logatchev 2006	12	7.09	4.73	0.70	0.66	0.064	0.076
Logatchev 2007	59	12.64	4.87	0.71	0.68	0.045**	0.039
Ashadzé	3	3.55	n.a.	n.a.	n.a.	n.a.	n.a.
South MAR	8	6.45	5.35	n.a.	n.a.	n.a.	n.a.

Number of individuals sampled (n), mean number of alleles across loci (*A*), *A_rich_* mean allelic richness, observed (*H*_O_) and expected (*H*_E_) heterozygosities and heterozygote deficiency (*F*_IS_) all obtained on the basis of 11 microsatellite loci used. Heterozygote deficiency (*F*_IS_) obtained using 7 microsatellite loci is also detailed. Significance levels are indicated (*p<0.05; **p<0.01 and ***p<0.001; bold numbers indicate significant values after q-value correction). n.a.- not applicable.

This study aims to infer present patterns of connectivity along the Mid-Atlantic Ridge (MAR) using higher resolution genetic markers and at a larger biogeographical scale than applied in any previous MAR studies in any species, and to determine whether the low genetic differentiation found among matrilines [22] also occurs at multiple biparentally inherited nuclear loci.

## Materials and Methods

### Sampling and DNA Extraction

Samples were collected from five locations corresponding to different hydrothermal vent fields (Rainbow, Logatchev, TAG, Ashadzé and South MAR) along the Mid-Atlantic Ridge (Table 1). For two locations (Rainbow and Logatchev), samples from different years were used to assess temporal variability; a variable time interval (Rainbow 1997, 1998, 2005, 2007 and 2008) and 3 consecutive one year intervals (Logatchev 2005, 2006, 2007) were used. Samples were collected using a slurp-gun from the ROV (Remotely Operated Vehicle) or the Nautile. Prior to each dive, the bowls used for collecting the shrimps were aseptically washed with ethanol (96%) before being filled with sterile seawater. Once on board, live specimens were either entirely frozen or immediately dissected into body components under sterile conditions and frozen or stored in 70% alcohol. DNA extraction was performed using the CTAB (cetyl trimethyl ammonium bromide) method [27] on muscle tissue. Sampling size could not be pre-defined to a constant value across all sampling sites and time steps due to the overall difficulty in obtaining samples from deep hydrothermal vents; in some sites sampling size was thus limited to the shrimp samples available from Ifremer research cruises for each particular location and time, and (for the 4°S location) some samples were kindly provided by the Max Plank Institute (N. Dubilier). The same samples have been used in a previous study with maternally inherited and lower resolution genetic markers [22].

**Table 3:** Mean allelic richness (*A_rich_*) calculated for the temporal samples.

	*A_rich_*					
Pop.\Year	1997	1998	2005	2006	2007	2008
Rainbow	8.98	8.96	7.99	n.a.	9.04	8.78
Logatchev	n.a.	n.a.	5.67	5.71	5.82	n.a.

Data shown are for a standardized minimum common sample size of 7 (Logatchev) or 19 (Rainbow) individuals.

### Microsatellite Genotyping

Microsatellite loci were amplified from the 687 individuals sampled, by PCR with 11 fluorescently-labelled forward primers (described in [28]). Each 10 µL reaction contained 10 ng of genomic DNA, 1x Qiagen HotStart *Taq* buffer, 200 µM of dNTP’s, 0.3 µM of each primer and 0.5 U of HotStart *Taq* polymerase (Qiagen). PCR amplifications were conducted on a Perkin-Elmer Gene Amp System 7200 (Waltham, MA, USA) with the following program: 15 min. at 95°C; 30 cycles composed of 30s at the annealing temperature [28], 30s elongation at 72°C and 30s of denaturation at 95°C, followed by 1 minute at the annealing temperature and a final 30 min. elongation step at 72°C. Fragments were separated on an ABI 3130 XL automatic sequencer (Applied Biosystems, Foster City, CA, USA) with the internal size standard Rox 350. Alleles were scored using Peak Scanner version 1.0 (Applied Biosystems).

**Figure 2:**
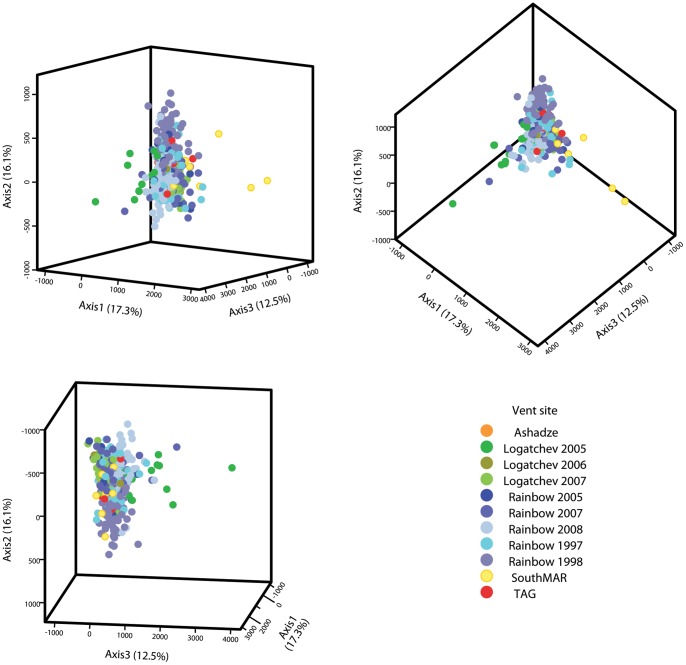
FCA plot. Three dimensional plot (presented from three different angles) of the factorial correspondence analysis for all sampled sites along the Mid-Atlantic Ridge and all sampling dates. Based on 11 microsatellite loci.

### Data Analysis

#### Genetic diversity and population differentiation

The mean number of alleles per locus (allelic diversity), the expected (*H*_E_) and observed (*H*_O_) proportion of heterozygotes, and the inbreeding coefficient (*F*_IS_) were estimated using the software GENETIX, 4.05 [29]. Significance levels were estimated using a permutation approach (1000 permutations). Linkage disequilibrium between all pairs of loci was tested for according to the Black & Krafsur [30] procedure, with 1000 permutations. Correction for multiple testing was performed using the false discovery rate (FDR) approach [31] in the software QVALUE [32]. For the temporal samples of the two populations, Logatchev and Rainbow, the rarefaction procedure implemented in GENCLONE [33] was used to calculate allelic richness (*A_rich_*), since the observed number of alleles in a sample is highly dependent on sample size.

**Table 4:** Pairwise *F*_ST_ between three sampled locations and years.

	Logatchev05	Logatchev06	Logatchev07	Rainbow97	Rainbow98	Rainbow05	Rainbow07	Rainbow08	TAG
Logatchev05		0.024	0.012	0.009	0.008	0.016	0.014	0.001	0.013
Logatchev06			0.007	0.002	−0.001	−0.006	−0.001	0.004	−0.007
Logatchev07				0.001	0.002	0.006	0.00004	0.002	0.003
Rainbow97					−0.001	0.002	−0.001	0.0002	0.001
Rainbow98						0.001	−0.0001	−0.0001	0.0002
Rainbow05							0.001	0.004	0.002
Rainbow07								0.0002	0.001
Rainbow08									0.004

None of the pairwise *F*_ST_ values were significant at the 5% level.

The *F* estimator of genetic structure θ [34] was calculated for each locus and over all loci. The probability of the *F*-statistics being greater than zero was determined by permutation (10 000 replicates) using GENETIX, 4.05 [29]. The genetic relationships across all genotyped individuals were also described by factorial correspondence analysis (FCA) using GENETIX; FCA detects the best linear combination of variables (allele frequencies at different loci) and describes the variation between observations. In all allelic frequency based analyses, only individuals from three sites (Rainbow, TAG and Logatchev) were used, whereas due to their low sampling size Ashadzé and SouthMAR were only used for individual based analyses.

**Table 5:** Estimation of *M* and θ generated in MIGRATE analysis of microsatellite data and mtDNA sequences (COI).

M	Microsatellite markers			mtDNA sequences		
Sites	Rainbow θ = 1.13	TAG θ = 1.16	Logatchev θ = 1.16	Rainbow θ = 3.7e−3	TAG θ = 2.9e−3	Logatchev θ = 4.3e−3
Rainbow	–	2.8	3.23	–	7.5e−12	9360
TAG	3.27	–	2.8	1.58e−10	–	2.27e−11
Logatchev	2.59	2.65	–	6990	21300	–

Donor populations are on vertical, recipient populations are on horizontal.

**Table 6:** Assessment of mutation model of 6 *R.exoculata* loci, assuming a single-step and a multi-step mutation model.

Locus	*p*	−2log*λ*	Mutation model
Rim 10	0.0835	33.77	TPM
Rim 19	0.01	0.336	SMM
Rim 23	0.0835	34.87	TPM
Rim 25	0.0345	7.78	TPM
Rim 31	0.016	2.02	SMM
Rim 32	0.0345	2.37	SMM

*p* indicates the proportion of multi-step mutations at max L(θ).

To investigate the number of distinct genetic populations represented by our sampling, we used the software STRUCTURE version 2.1 [35]. All runs were based on an initial burn-in of 50 000 cycles with 100 000 additional cycles, and runs were iterated 6 times for each K, from 1 to 11. Both Pritchard’s [35] L(K) criterion and the ΔK criterion of Evanno *et al.* [36] were used to verify consistency across methods. In all simulations, an admixture ancestry model and correlated allele frequency model were used.

#### Inference of connectivity and population effective size

To assess asymmetrical gene flow between the different hydrothermal vents, we used the software package MIGRATE version 3.2.16 [37]. This analysis is based on maximum-likelihood (ML) estimates for both migration rates and effective population sizes using a coalescent approach [38]. To avoid the confounding effects of differences in sample size, we randomly resampled individuals from the larger group, to reach the same sample size as in the smallest group. We used a Brownian motion model, an initial random seed number and θ and M starting parameters calculated from *F*_ST_. As searching strategy we used 10 short chains (1000 trees sampled) and three long chains (10 000 trees sampled). For each chain the first 100 000 steps were used as a burn-in and adaptive heating was used to ensure an independent, comprehensive search of the parameter space. In order to directly compare some of the results obtained in this study using microsatellite markers, and the previous results obtained with a mitochondrial marker [22] we re-analyzed the former mitochondrial data. We ran MIGRATE four times on each of the two molecular datasets to verify the consistency of results, and used the average of the estimates obtained in each run.

**Table 7:** Results of bottleneck tests for the spatial and temporal samples of *R. exoculata*.

	P (H deficiency)	
Population	SMM	TPM
Rainbow 1997	0.02	0.02
Rainbow 1998	0.008	0.008
Rainbow 2005	0.008	0.008
Rainbow 2007	0.008	0.02
Rainbow 2008	0.008	0.008
TAG	0.008	0.02
Logatchev 2005	0.02	0.04
Logatchev 2006	0.04	0.04
Logatchev 2007	0.008	0.02

To assess the possible site of origin of the individuals from Ashadzé and South MAR, assignment/exclusion analyses were performed using GENECLASS2 [39]. These assignment tests were also used to infer potential migration, by analyzing all other individuals. We used the partial Bayesian classification method [40] implemented in GENECLASS2, paired with a Monte Carlo re-sampling method for computation of assignment probability to each population [41], using 10 000 simulated individuals. To determine the assigned and unassigned individuals we considered that if the probability of assignment of an individual was >0.05 in only one population, it was considered to be a resident of that population. If its probabilities of assignment exceeded 0.05 in more than one population, it was left unassigned, finally, if its probabilities of assignment were <0.05 in all populations, it was considered to be an immigrant from outside the sampled area [42].

#### Demographic stability

To test for a reduction in effective population size linked to bottleneck or founder events, the Wilcoxon sign-rank test was applied to infer if expected heterozygosities estimated from allele frequencies (*H*_E_) were higher than estimates based on the number of alleles and sample size (*H*_Eq_). During a bottleneck, allele number decreases faster than heterozygosity, resulting in a transient apparent heterozygosity excess, indicative of a recent bottleneck event [43] whereas the opposite (allele excess) might occur during a population expansion [44]. Tests were implemented by BOTTLENECK 1.2.02 [43] using 1000 iterations. Estimates of *H*_Eq_ were calculated under the single-step mutation model (SMM) and the two-phase model (TPM), allowing for i) 10% and 2) 4% of multi-step mutations. The latter TPM model was chosen after assessing the mutation model of the microsatellite markers using a likelihood approach implemented by MISAT [45]. The program MISAT calculates the likelihood of the data given a single-step mutation model (SMM), or alternatively a two-phase model that allows a proportion of mutations (*p*) to involve changes greater than a single repeat. Model fit was then statistically tested by the differences in likelihoods. For each locus, Markov chains were run for 100,000 generations for SMM and TPM models, and *p* in the TPM was allowed to vary between 0.0001 and 0.5. Likelihood ratio tests were used as described in [45], by calculating −2logλ, where λ = [max likelihood of single-step model/max. likelihood of multi-step model], we conclude one model fits the data significantly better than the other by comparing −2logλ to the χ^2^ distribution (d.f. = 1).

To characterize past changes in the effective population size (*N*_e_) of *R. exoculata* we generated Bayesian skyline plots using mitochondrial data [46] with Beast v.1.5.4 [47]. We used the mutation rate of 0.7% per Myr based on [48] for other caridean shrimp. Bayesian skyline plots generate a posterior distribution of *N*_e_ through time using Markov chain Monte Carlo (MCMC) sampling. The reconstructed Bayesian skyline plots (BSP) were obtained using the HKY model as selected by the AIC criterion implemented in Modeltest [49]. A constant population size coalescent model was selected. All analyses ran for 200 million iterations with the first 10% discarded as burn-in. Genealogies and model parameters were sampled every 2 000 iterations. Bayesian skyline reconstructions were conducted in Tracer v.1.5 [47] to determine the effective population size over time (*N*_e_T), the median and the corresponding credibility intervals.

### Ethics Statement

No specific permits were required for the described field studies. No specific permissions were required for these locations/activities. The sampled locations are not privately-owned or protected in any way, and the field studies did not involve endangered or protected species.

## Results

### Genetic Diversity

Multilocus genotypes from 687 shrimp were obtained from the five hydrothermal vent fields along the Mid-Atlantic Ridge. The number of alleles per locus varied from 7 (loci Rim19 and Rim25) to 52 (Rim26) over all locations (Fig. 1). The mean number of alleles per locus ranged from 3.55 (Ashadzé, n = 3) to 17.91 (Rainbow 1998, n = 166), increasing with sample size, as expected. Mean allelic richness (*A_rich_*) standardized for comparison across a minimum common sample size of 5 individuals (Ashadzé site was excluded from these calculations), ranged from 4.52 (Rainbow 2005) to 5.35 (SouthMAR). No loci showed evidence of linkage disequilibrium. Unbiased heterozygosity (*H*_E_) varied between 0.66 (Rainbow 2005) and 0.71 (Logatchev 2007) and the observed heterozygosity (*H_O_*) varied between 0.58 (Rainbow 2005) and 0.68 (Logatchev 2007) for the three sampled sites (Rainbow, TAG and Logatchev) and years (Table 2). Four loci were identified as possibly having technical issues (null alleles and/or large allele dropout), since they presented high *F*_IS_ for most populations. To ensure that these loci were not introducing error estimations, all analyses (*F*_IS_ and pairwise *F*_ST_ comparisons) were repeated without the 4 possibly problematic loci resulting in the same trends as obtained with all 11 loci (Table 2). Using the 11 markers, the tests for Hardy-Weinberg equilibrium revealed one site with significant departure (heterozygote deficiency) after correction for multiple tests (Table 2): Rainbow (all sampled years). However, removing the 4 loci suspected to have null alleles leaves only samples from two time steps in Rainbow (1997 and 1998) with significant departure from Hardy-Weinberg equilibrium (Table 2).

At a temporal scale of three consecutive years (2005–2007), the Logatchev vent did not show significant differences in genetic diversity between years, neither in *H_O_* (Table 2) nor mean allelic richness (Table 3). For the Rainbow vent, with samples spanning 11 years, observed heterozygosity (*H_O_*) varied from 0.58 (2005) to 0.66 (2007) across years (Table 2) and mean allelic richness in this vent also varied from 7.99 (2005) to 9.04 (2007) using a minimum size of 19 diploid individuals (Table 3).

### Population Structure, Connectivity and Demography

Pairwise *F*_ST_ estimates between vent locations (Table 4) were not significantly different from zero (P>0.05), after q-value correction for multiple tests. The FCA analysis based on allelic frequencies (Fig. 2) demonstrates along the first axis (17.3%) a slight differentiation of the South MAR vent from the other vents; however the overall analysis demonstrates a lack of structure among the 5 vents. A similar pattern was detected using the STRUCTURE analysis, where only one cluster (mean L(K = 1) = −26197, all others: mean L(K = 2−11)>−26405) was detected with both L(K) and ΔK methods (data not shown).

Estimates of gene flow obtained with MIGRATE software ranged from 2.59 (Logatchev to Rainbow) to 3.27 (TAG to Rainbow) with no apparent asymmetrical migration between the three hydrothermal vent sites (Table 5). In agreement with microsatellite data, MIGRATE results using mitochondrial data (Table 6) did not reveal a clear northward or southward direction of gene flow. The results of the assignment/exclusion analysis with GENECLASS2 software also supported panmixia at the ridge level, with a percentage of unassigned individuals of 99.4% due to high probabilities of assignment to more than one site.

The estimation of the mutation model of 6 microsatellite marker using MISAT revealed that 3 microsatellites followed a SMM model and 3 others a TPM with multi-step mutations with a rate ranging from 3.45% to 8.35% (Table 6). For the five remaining markers the model could not be assessed due to low likelihood scores.

The bottleneck tests indicated a significant recent expansion in effective population size along the Mid-Atlantic Ridge (using all mutation models tested), as the expected heterozygosity estimated from allele frequencies (*H*_E_) was lower than estimates based on the number of alleles and sample size (*H*_Eq_). The Wilcoxon sign-rank results revealed significant heterozygosity deficiency for all sites and years sampled (Table 7).

**Figure 3:**
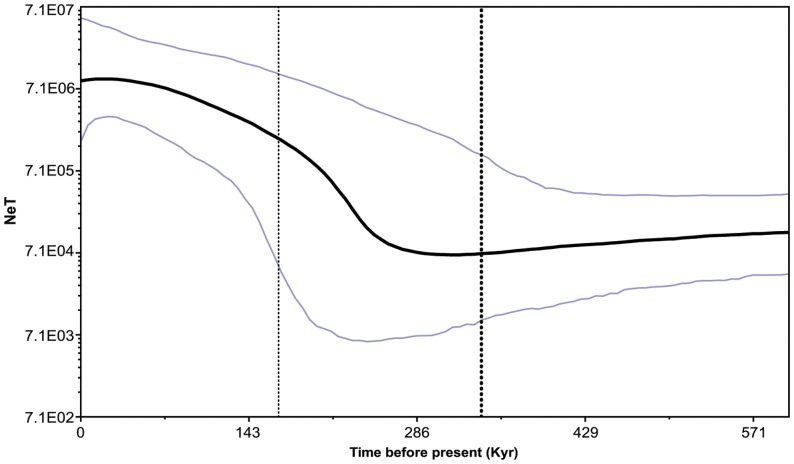
Bayesian skyline plot. Graph depicting changes in effective population size over time based on mitochondrial COI sequence data of *Rimicaris exoculata*, and based on a mutation rate of 0.7% (this mutation rate may be overestimated for a deep-sea vent species when compared to coastal ones). On the y-axis *N*_e_ represents effective population size and T is generation time; on the x-axis time is represented in thousands of years (Kyr). The thick black line represents the median and the blue lines the 95% highest posterior density of the *N*_e_T estimates.

The resulting output of the MCMC analysis using BSP is summarized in Figure 3, indicating the variation in *N*_e_ during the last 600 Kyr, this variation with time is based on a 0.7% mutations per million years, under this scenario of assumed mutation rate there is an increase in population effective size of *R. exoculata* that is placed around 250 Kyr ago.

## Discussion

This study revealed a lack of genetic structure in a hydrothermal vent endemic species, the shrimp *Rimicaris exoculata,* along the 7100 Km of the Mid Atlantic Ridge. The large scale panmixia and probable recent demographic fluctuations in *Rimicaris exoculata* along the Mid-Atlantic Ridge, here inferred using microsatellites, are congruent with the results obtained from mtDNA analysis [22]. The use of eleven microsatellite loci, allow the interpretation of these observations as the result of neutral processes rather than originating from selective sweep, process that could not be strictly discarded on the basis of the mitochondrial locus alone.

A lack of power due to low genetic variation can also be discarded as an alternative hypothesis, as high genetic diversity was detected within all studied vents. Both the mean number of alleles per locus (3.55–17.91) and gene diversity (0.66–0.71) are within the values found for other hydrothermal vent organisms (e.g. [12], [50]). However, in another hydrothermal vent shrimp species, *Chorocaris* sp, similar allelic richness (2–16) and higher variability in gene diversity (0.17–0.82) were detected using microsatellite markers, on the basis of a much smaller sample size [51]. Along the Mid-Atlantic Ridge, microsatellite markers have only been used on one species, the commensal polychaete *Branchipolynoe seepensis* [12], revealing a high variation in genetic diversity (gene diversity 0.16–0.94) for the three studied vent sites (Lucky Strike, Broken Spur and Logatchev [12]).

A scenario of current persistent large-scale effective dispersal (gene flow) is indicated by the lack of spatial genetic differentiation estimated from the non-significant pairwise differentiation, the high gene flow estimates and the inference of one single population in the MAR. The different demographic analyses based on either microsatellites or DNA markers support a recent expansion, possibly associated to a common (re)colonization event, about 250 Kyr ago (estimates from mtDNA Bayesian Skyline plots, under the hypothesised mutation rate of 0.7% [48]). This mutation rate might however be overestimated, since it is based on coastal caridean shrimp, whereas deep-sea species might have slower mutation rates [52]. We are not aware of any significant unique geological or climatic records that could explain a change in vent activity or water column conditions that may have modified at once the habitat conditions for adults and/or larvae. Whether a dramatic change in vent activity along MAR occurred around this time could be tested for in the future by performing similar analysis on other species sharing a similar distribution as *R. exoculata*, yet exhibiting distinct larval development in relation to vertical migration in the water column.

Together with the spatial and demographic analyses, the trends in temporal variability also revealed no genetic differentiation among samples separated by one to a maximum of eleven years for two vent sites (Logatchev and Rainbow). These results may indicate large effective population sizes (*N*_e_) at these demes and/or at the scale of the metapopulation, therefore rendering the effect of genetic drift negligible over the temporal scales considered in this study. Two scenarios may explain the large scale and temporal panmixia, i) present day large-scale effective dispersal and/or ii) an historical event of extinction followed by (re)colonization of all vents from a common source, where insufficient time elapsed since the (re)colonization event, compared to the likely large effective population size, rendering the effects of genetic drift negligible [53]. Both scenarios support the existence of effective large-scale dispersal of *Rimicaris exoculata* along the MAR. This large-scale dispersal was not shown to exhibit directionality or source-sink relationships between the studied sites. This is in agreement with the few studies available on circulation along the MAR, the ridge being constrained by very high walls that flank its axial valleys [54], where in general currents show high dependence on the topography of each site, resulting in residual currents at different hydrothermal vent sites along the Mid-Atlantic ridge having sometimes opposite directions [55].

Our results show that these highly fragmented and specialized environments may paradoxically be considered for some species as open systems with free exchange of planktonic larvae or adults. Such functioning has long been a paradigm when considering marine systems [56], and has been severely questioned during the last decade in shallow water ecosystems after repeated reports of genetic structure despite a lack of obvious physical barriers [57]. The observation of such an open system in a highly fragmented and specialized deep sea ecosystem is therefore puzzling. Of all species studied to date on hydrothermal vent habitats, *R. exoculata* is the first to exhibit no barriers to dispersal across such a high geographic extent. Within the EPR region, some species have been shown to exhibit large scale dispersal, however in all a limitation to dispersal at the scale of several hundreds to thousands of kilometers was found, due to either facture zones [8] or isolation by distance [58].

Besides showing homogeneity over shorter distances, these species from the EPR exhibit different life history traits at the egg and larval stage, which in different ways might potentially increase dispersal ability. These include free-swimming planktotrophic larvae (in the mussel species *Bathymodiolus thermophilus* [59]), large lecitotrophic eggs (in the commensal scaleworm *Branchipolynoe symmytilida* [60]) and lecitotrophic larvae able to arrest development in cold bottom waters (in the polychaete *Alvinella pompejana* [61]), all of which are expected to contribute to prolonged larval duration (PLD) and therefore higher dispersal potential. However, a species with non-swimming lecitotrophic larvae (the polychaete *Tevnia jerichonana*), expected to reduce dispersal ability, has been reported to have high gene flow along part of the East Pacific Rise [62]. In comparison with these cases in the Pacific, the Mid Atlantic Ridge would be expected to impose greater limitations on dispersal. This is due to larger distances (at least 100 km) and greater depth variation among active fields, slower spreading rate and larger transform offsets and reliefs [1], [8], rendering more unexpected the lack of population structure here found in shrimp along the deep Atlantic.

*Rimicaris exoculata* larvae go through a long planktotrophic phase suspected to partly take place in the photic zone due to the photosynthesis-derived lipids [63] isolated in new recruited juveniles. These findings suggested that larvae achieve long-distance dispersal (vertical followed by horizontal migration) via the upper layers of the water column [18], although the vertical migration of the larvae might also have the sole purpose of feeding in the plankton and providing an ontogenic development in adequate conditions of temperature and pressure [64]. Many postlarvae were found close to the bottom of a MAR hydrothermal vent (Broken Spur; [65]) and a remarkable disparity in the relative abundance of adults and pelagic postlarvae at the same site, led these authors to suggest that the postlarvae found at Broken Spur originated elsewhere. These results support the larval stage as the means for long-distance dispersal, although adult swarms might also play a role.

Three-dimensional trajectories may be aided by mesoscale eddies which can affect the seafloor at great depths and potentially transport vent-associated organisms for hundreds of kilometers [66]. Such processes might enable dispersal by *R. exoculata* to be unaffected by seafloor barriers, but our discovery of large scale effective dispersal across the immensity of oceanic masses still allowing successful recruitment at their rare and fragmented habitat seriously questions the hypothesis of purely passive dispersal.

This study challenges the classical view of passive large scale dispersal subject to haphazard trajectories of currents in different layers of the water column. In the three dimensional ocean, the probability of an individual reaching a suitable habitat after being diluted in an incommensurable volume of water seems infinitesimal and calls for the existence of non-passive, dispersal strategy mechanisms such as those delaying metamorphosis or actively guiding larvae or adults towards suitable habitat. Recent studies showed that planktonic larval duration is strongly influenced by water temperature for many fish and invertebrate taxa [67]. The very low temperature between vent sites, contrasting with the temperature at their hydrothermal habitats, might delay metamorphosis until its activation by a trigger, possibly linked to the extreme physical or chemical characteristics of vent sites, recalling temporal dispersal (e.g., seed or cyst dormancy) of unrelated organisms. A hypothesis of active directed migration could involve the detection of stimuli such as water chemistry, sound, polarized light, current direction, magnetism and water pressure, as found for other organisms [68]. *R. exoculata* adults have been shown to possess a dorsal photoreceptor that is highly sensitive to black body radiation associated with high-temperature vents [69], with chemoreceptors allowing to detect picomolar concentrations of H_2_S [70]. Such possible vent tracking mechanisms have been demonstrated for adults at scales of meters. The spatial extent of their influence or their complementarities with larger scale mechanisms such as hyper-delayed metamorphosis, remain to be determined. In any case, our results challenge the view of passive dispersal, by revealing widespread connectivity in deep-sea taxa inhabiting discrete and distant habitats. This calls for re-appraisal of the role of physiological adaptations for effective dispersal on the ecology and evolution of marine organisms.
